# In Vivo Bioavailability of Selenium in Selenium-Enriched *Streptococcus thermophilus* and *Enterococcus faecium* in CD IGS Rats

**DOI:** 10.3390/antiox10030463

**Published:** 2021-03-16

**Authors:** Gabriela Krausova, Antonin Kana, Marek Vecka, Ivana Hyrslova, Barbora Stankova, Vera Kantorova, Iva Mrvikova, Martina Huttl, Hana Malinska

**Affiliations:** 1 Department of Microbiology and Technology, Dairy Research Institute, Ltd., Ke Dvoru 12a, 160 00 Prague, Czech Republic; hyrslova@milcom-as.cz (I.H.); mrvikova@milcom-as.cz (I.M.); 2 Department of Analytical Chemistry, Faculty of Chemical Engineering, University of Chemistry and Technology Prague, Technicka 5, 166 28 Prague, Czech Republic; Antonin.Kana@vscht.cz (A.K.); Vera.Kantorova@vscht.cz (V.K.); 3 4th Department of Medicine Department of Gastroenterology and Hepatology, First Faculty of Medicine, Charles University in Prague, U Nemocnice 2, 128 00 Prague, Czech Republic; marek.vecka@lf1.cuni.cz (M.V.); barbora.stankova@lf1.cuni.cz (B.S.); 4 Centre for Experimental Medicine, Institute for Clinical and Experimental Medicine (IKEM), Videnska 1958/9, 140 21 Prague, Czech Republic; martina.huttl@ikem.cz (M.H.); hana.malinska@ikem.cz (H.M.)

**Keywords:** selenium-enriched *Enterococcus faecium*, selenium-enriched *Streptococcus thermophilus*, antioxidant capacity, glutathione reductase, glutathione peroxidase, CD IGS rats, oxidative stress, lactic acid bacteria

## Abstract

The selenium (Se) enrichment of yeasts and lactic acid bacteria (LAB) has recently emerged as a novel concept; the individual health effects of these beneficial microorganisms are combined by supplying the essential micronutrient Se in a more bioavailable and less toxic form. This study investigated the bioavailability of Se in the strains *Enterococcus faecium* CCDM 922A (EF) and *Streptococcus thermophilus* CCDM 144 (ST) and their respective Se-enriched forms, SeEF and SeST, in a CD (SD-Sprague Dawley) IGS rat model. Se-enriched LAB administration resulted in higher Se concentrations in the liver and kidneys of rats, where selenocystine was the prevalent Se species. The administration of both Se-enriched strains improved the antioxidant status of the animals. The effect of the diet was more pronounced in the heart tissue, where a lower glutathione reductase content was observed, irrespective of the Se fortification in LAB. Interestingly, rats fed diets with EF and SeEF had higher glutathione reductase activity. Reduced concentrations of serum malondialdehyde were noted following Se supplementation. Diets containing Se-enriched strains showed no macroscopic effects on the liver, kidneys, heart, and brain and had no apparent influence on the basic parameters of the lipid metabolism. Both the strains tested herein showed potential for further applications as promising sources of organically bound Se and Se nanoparticles.

## 1. Introduction

In recent years, natural antioxidants have attracted interest from researchers because of the diseases caused by free radicals and reactive oxygen species (ROS), including heart disease, atherosclerosis, neurological disorders, and type II diabetes. The World Health Organization (WHO) expects cardiovascular disease to be the leading cause of mortalities by 2030, affecting approximately 23.3 million people [1]. Enrichment of the diet with antioxidants is a basic prerequisite for maintaining good health. In addition to synthetic antioxidants, natural antioxidants, including probiotic microorganisms and their metabolic products, are also available. Probiotic bacteria are defined as living microorganisms that, when consumed in sufficient quantities, confer health benefits to the host, such as the modulation of the immune system, maintenance of healthy intestinal microbiota, and lowering of cholesterol or prevention of diarrhea [2]. Probiotic microorganisms can act as antioxidants in several ways, such as by trapping ROS, chelating metal ions, inhibiting enzymes, and reducing or inhibiting ascorbate autooxidation. Furthermore, the metabolic activities of probiotics can have antioxidant effects, such as the capturing of oxidative compounds or prevention of their formation in the intestine [3]. One of the ways to increase the antioxidant activities of probiotic strains is their enrichment with selenium (Se), an essential microelement for humans found in a number of protein-based enzymes, including glutathione peroxidases, thioredoxin reductases, and iodothyronine deiodinases; in humans, these enzymes are associated with the responses to oxidative stress resulting from various biological activities [4]. The antioxidant enzymes glutathione peroxidase (GPx), glutathione (GSH), and superoxide dismutase (SOD) protect cells from oxidative damage by eliminating superoxide-free radicals [5]. Malondialdehyde (MDA), formed during lipoxidation, indicates cell or tissue injury and is an indicator of lipid peroxidation [6]. Furthermore, Se supplementation has beneficial effects in cancer, inflammation, immune responses, cardiovascular disease, thyroiditis, and male fertility [7]. On the contrary, low Se levels have been associated with an increased risk of mortality, poor immune function, and cognitive decline [8]. Se is involved in the immune responses of the body [9,10], similar to other natural substances such as probiotics. The positive effects of probiotics and Se co-supplementation on human mental health, hormonal profiles, and biomarkers of inflammation and oxidative stress have been documented [5,11,12]. For example, Se-enriched *Bifidobacterium longum* has a hepatoprotective effect in mice fed a high-fat diet [9]. Furthermore, Se-enriched probiotics exhibit antibacterial activity against pathogenic *Escherichia coli* [13]. The selenization of probiotics and lactic acid bacteria (LAB), a recently suggested concept, may provide multiple health benefits to the host. The selenization of yeasts and LAB confers benefits such as the following: (i) the delivery of Se for biological processes; (ii) increased bioavailability of Se in its less toxic organic form; (iii) individual health benefits of LAB and yeasts in live or inactivated forms (e.g., organic acid production, probiotic function, production of antimicrobial compounds, bacteriocins, etc.); and (iv) less burden on the environment due to the organic form of Se.

Selenized strains of yeasts and LAB represent sources of the more bioavailable organic forms of Se, making them more suitable application forms compared to food supplements containing Se in its inorganic form, such as sodium selenite [14]. Additionally, there is evidence suggesting that selenized cells of some LABs show a higher resistance to various stress conditions compared to the parental cells [15,16,17].

Se deficiency is as dangerous as its overdose. Despite the recognition of Se as an important microelement, discussion on the mode of its supplementation remains controversial. The main concern of Se supplementation is selenium nephrotoxicity, because this element accumulates in the kidneys, liver, and respiratory organs [18]. The most toxic forms of Se are the inorganic species SeIV and SeVI, which are permitted as food supplements in the form of sodium selenite, sodium hydrogen sulfite, and sodium selenate by Commission Regulation No. 1170/2009. However, selenium-enriched yeast containing this element in the organic form is also permitted for use in foodstuffs and food supplements under this regulation in the European Union. Some strains of LAB, bifidobacteria, and yeast can biotransform inorganic selenium present in the growth medium [19] into organic forms such as selenocysteine, selenomethionine, or methylselenocysteine [20,21]. Additionally, probiotics containing organic selenium species have shown negligible liver and kidney toxicity [18]. To obtain products that are well-defined in terms of health benefits and, more importantly, the safety of consumers, it is necessary to map selenium metabolism, its accumulation, and biotransformation in each Se-enriched strain individually.

In our previous study [22], an in vitro assessment of the properties of the selenized LAB strains *Streptococcus thermophilus* CCDM 144 and *Enterococcus faecium* CCDM 922A revealed that both strains could tolerate sodium selenite and accumulate, as well as biotransform Se into its organic form. These strains were further studied for their beneficial properties and potential to improve the host health. As previously reported [23], the strain CCDM 922A exhibits immunomodulatory abilities and the capacity to reduce low-density lipoprotein and very low-density lipoprotein cholesterol in rats. Good adhesion properties and the presence of the gene encoding the bacteriocin enterocin A were also observed in the strain CCDM 922A. *Streptococcus thermophilus* CCDM 144 is an exopolysaccharide (EPS)-producing strain used in the production of fermented dairy products, as part of the yogurt culture, to improve their texture. Both strains are commercially used in dairy processing plants, and the CCDM 922A strain is also used in animal nutrition. However, additional information is needed to evaluate the safety, functionality, and properties of their selenized forms. Overall, further studies are required to obtain legislative approval for the use of selenized LAB in food and food supplements. Hence, with the intention to jumpstart this process, this study aimed to evaluate the effects of the two promising Se-enriched candidates of LAB, the strains *S. thermophilus* CCDM 144 and *E. faecium* CCDM 922A, on the biological functions of rats in vivo.

## 2. Materials and Methods

### 2.1. Bacterial Strains and Their Selenium Enrichment

The two LAB strains, *S. thermophilus* CCDM 144 (ST) and *E. faecium* CCDM 922A (EF), used for selenization in this study were provided by the Culture Collection of Dairy Microorganisms Laktoflora^®^ (Tábor, Czech Republic). M17 broth and M17 agar, according to Terzaghi (Merck, Darmstadt, Germany), were used for precultivation and storage. Both strains were cultured under aerobic conditions at 37 °C for 48 h. For enumeration of the bacterial cells, 10-fold dilutions of the cultures were plated, and the resultant colony-forming units were counted. For selenization, 50-mg/L sodium selenite (Sigma-Aldrich, Steinheim, Germany) was added to the M17 broth, and fresh overnight grown cultures of each strain were inoculated into the M17 medium at a final concentration of 10^5^ colony-forming units (CFU)/mL and cultivated for 24 h at 37 °C under aerobic conditions. As controls, the strains CCDM 144 and CCDM 922A were grown in selenite-free M17 cultivation medium. After cultivation, the cells were centrifuged repeatedly at 4100× *g* for 10 min (Spectrafuge 6C, Labnet International Inc., Edison, NJ, USA) and washed twice with sterile saline to remove unbound selenium. Subsequently, 1% inoculum of each bacterial suspension was added to skimmed (1.5% fat) and Ultra-heat treatment (UHT)-treated milk and incubated aerobically at 37 °C for 16–18 h. The pH was adjusted to 5.5–6.0 using 15% NaOH. Following this, the obtained samples were lyophilized (Lyobeta 35; Telstar, Barcelona, Spain) with the addition of 20% lactose as the cryoprotective medium, and the total amount of Se bound to the bacterial cells and Se species were determined in the samples. In this manner, the Se-enriched biomass of both strains, SeST and SeEF, were used for further application as foodstuffs. The final counts (colony-forming units (CFU)) of the strains EF, ST, SeEF, and SeST after lyophilization were also determined.

### 2.2. Animal Model and Study Design

All experiments with laboratory animals were conducted in compliance with the laws of the Czech Republic (311/1997 column.) and the European Community Council recommendations (86/609/EEC) regarding the protection of animals used for experimental and other scientific purposes. This experimental study was approved by the committee of the Ministry of Education, Youth, and Sports of the Czech Republic, approval No. MSMT-11197/2020-2. A total of 48 adult cesarian derived (CD) (SD-Sprague Dawley) International Genetic Standardization (IGS) male rats (Velaz, Prague, Czech Republic)—outbred multipurpose breed of albino rat—were used in this study. Six-week-old rats were randomly divided into six groups (A–F) (*n* = 8) and fed a standard maintenance diet and tap water for two weeks to acclimatize. Rats were housed in cages in a room with controlled temperature (22–24 °C), humidity (55–60%), and natural light conditions (12-h light/dark cycle). Following acclimatization, the experimental diets were administered to the animals for 58 days. Group A was fed a standard maintenance diet of Altromin 1324, control group B was fed a Se-deficient diet of Altromin C 1045, group C was fed Altromin C 1045 with added *S. thermophilus* (ST), group D was fed Altromin C 1045 with added selenized *S. thermophilus* (SeST), group E was fed Altromin C 1045 with added *E. faecium* (EF), and group F was fed Altromin C 1045 with added selenized *E. faecium* (SeEF).

Twenty-two grams of the appropriate diet was administered daily to the animals. Tap water was supplied ad libitum. After 58 days, rats were euthanized by decapitation following anesthetization (zoletil 5 mg/kg body weight) in the postprandial state. Aliquots of the serum and tissue samples were collected and stored at −80 °C for further analyses.

### 2.3. Experimental Diets

The following six types of diets were administered to the rats: (a) standard maintenance diet of Altromin 1324 (Altromin Spezialfutter GmbH & Co, Lage, Germany), (b) Altromin C 1045 as the Se-deficient diet (control group), (c) Altromin C 1045 with added *S. thermophilus* CCDM 144 (ST), (d) Altromin C 1045 with added selenized *S. thermophilus* CCDM 144 (SeST), (e) Altromin C 1045 with added *E. faecium* CCDM 922A (EF), and (f) Altromin C 1045 with added selenized *E. faecium* CCDM 922A (SeEF). All diets were processed into 10-mm pellets. The special diets were manufactured by Altromin Spezialfutter GmbH & Co, and the lyophilized strains ST, EF, SeST, and SeEF in the milk matrix were processed into the feedstuff by the manufacturer. Based on the measurements of the total Se concentrations in the lyophilized matrices, the dosage in the feed mixture was determined. Detailed information about the Se content and its chemical form in the individual diets is presented in Table 1. The Se concentrations used in this study were safe in terms of toxicity, as the diets of groups D and F, which were supplemented with selenized strains, had only slightly higher Se concentrations than that in the standard maintenance diet.

### 2.4. Antioxidant and Oxidative Stress Parameters

Blood samples were collected gravitationally following the decapitation of the rats, and the serum was isolated from the blood by centrifugation at 1500× *g* at 4 °C for 10 min. Tissues were removed and immediately weighed, washed with cold phosphate-buffered saline (PBS) (0.02 mol/L, pH 7.1), and aliquoted in PBS (300–500-mg tissue in 0.5-mL PBS; 0.6–1.0 g in 1-mL PBS). The resulting suspension was subjected to two freeze/thaw cycles. The homogenate was centrifuged (1500× *g* for 15 min), and the supernatant was divided into three minimal aliquots, which were stored at −80 °C. Concentrations of total cholesterol, high-density lipoprotein (HDL) cholesterol, and triacylglycerols were assessed by enzymatic-colorimetric methods (CHOD/PAP, direct homogeneous enzymatic-colorimetric reaction without precipitation, GPO/PAP; Lab Mark a.s., Prague, Czech Republic) using an automatic biochemical analyzer modular (Roche, Prague, Czech Republic). The concentrations and activities of the antioxidants glutathione peroxidase (GPx), glutathione reductase (GR), and malondialdehyde (MDA) in the serum, brain, and heart tissue were evaluated by the sandwich heterogenous enzymatic immunoassay using commercial Enzyme-Linked ImmunoSorbent Assay (ELISA) kits for rat GPx, GR, and MDA (MyBioSource Inc., San Diego, CA, USA). Lipoperoxidation products were assessed based on the levels of thiobarbituric acid-reactive substances (TBARS) by assaying the reaction with thiobarbituric acid.

### 2.5. Determining Total Selenium and Se Species Contents

The total contents of Se, Cu, Zn, Cr, and Fe in the liver and kidneys, as well as in the feedstuff, were determined using inductively coupled plasma mass spectrometry (ICP-MS) after microwave-assisted acid digestion. Three microliters of concentrated nitric acid (67% Analpure^®^, Analytika spol. s r.o., Prague, Czech Republic) was added to the homogenized feedstuff and tissue samples that were placed in a Teflon^®^ digestion vessel (Berghof, Germany). The mixture was then mineralized in a closed vessel in a microwave digestion system (Speedwave 4; Berghof, Germany) for 15 min at 200 °C. After cooling, the decomposed samples were transferred to 50-mL volumetric flasks, spiked with rhodium solution (Astasol^®^, Analytika spol. s r.o., Czech Republic) as an internal standard to obtain a final concentration of 20-µg/L Rh after adjusting the volume with demineralized water (Milli-Q, Millipore, Billerica, MA, USA). The ICP-MS (DRC-e; Perkin-Elmer, Concord, ON, Canada) measurement conditions were as follows: RF (radio-frequency) power, 1.4 kW; nebulizer gas flow rate, 0.76 L/min; auxiliary gas flow rate, 1 L/min; plasma gas flow rate, 11 L/min; and measured isotopes ^80^Se, ^63^Cu, ^66^Zn, ^53^Cr, ^57^Fe (analytes), and ^103^Rh (internal standard). The spectral interference of ^40^Ar_2_^+^ was eliminated using a dynamic reaction cell with methane as the reaction gas (methane flow rate 0.3 mL/min, rejection parameter *q* 0.3).

The selenium species were identified using ion-pair liquid chromatography (HPLC) coupled with ICP-MS, as described previously [22]. Briefly, reversed-phase column (Purospher^®^ STAR RP-8 endcapped, 250 × 4.6 mm, 5 µm, Merck, Germany) and the mobile phase consisting of 1.0-g/L sodium butane-1-sulfonate, 0.22-g/L tetramethylammonium hydroxide pentahydrate, 0.42-g/L malonic acid, and 0.05% (*v*/*v*) methanol (Sigma Aldrich, Steinheim, Germany) were used for Se species separation. To extract the samples by enzymatic hydrolysis, 0.25 g of the tissue sample or 0.1 g of the feedstuff sample was weighed in 15-mL polypropylene tubes and homogenized for 1 min at 20,500 rpm using a disperser Ultra-Turrax^®^ with 2 mL of 20-mmol/L tris-(hydroxymethyl)-aminomethane (Fluka, Buchs, Switzerland) solution buffered (pH = 7.0) using HCl (Suprapur^®^, Merck, Germany). Thereafter, 25 mg of protease XIV (Sigma-Aldrich, Tokyo, Japan) was added, and the sample was extracted for 18 h at 37 °C. The reaction mixture was then filtered through a 0.45-µm syringe nylon filter (Whatman, Maidstone, UK) and analyzed. The standards of the selenium species, selenate (SeVI), selenite (SeIV), selenomethionine (SeMet), selenocystine (SeCys2), and Se-methylselenocysteine (MeSeCys) were obtained from Sigma Aldrich (Germany). Calibration solutions of the selenium species containing 1-, 5-, 10-, 50-, and 100-μg/L Se were prepared by diluting the stock solutions of the selenium species with water.

### 2.6. Detection of Se Nanoparticles Using Transmission Electron Microscope (TEM)

Images of nanoparticles (NPs) were acquired using a TEM Jeol 2200 FS (Jeol, Tokyo, Japan). Elemental mapping of the selected region was conducted using an energy-dispersive X-ray spectrometer (EDX) attached to the TEM. The TEM images were evaluated using ImageJ 1.50i software (Wayne Rasband, National Institutes of Health, College Park, MD, USA). Fresh bacterial suspensions of the strains CCDM 144 and CCDM 922A grown overnight in M17 broth supplemented with 10 mg/L of sodium selenite were centrifuged (6000× *g*, 5 min) and resuspended in water. Samples for TEM imaging were prepared by evaporating (3 h at 24 ± 1 °C) a drop of culture placed onto a 300-mesh lacey carbon copper grid.

### 2.7. Microbiota Composition

Samples of the distal part of the colon with feces were taken into sterile samplers containing Wilkins-Chalgren Anaerobe broth (Oxoid Limited, Basingstoke, Hampshire, UK) and frozen at −20 °C immediately after collection until further analyses. Differences in the composition of the gut microbiota among the experimental groups were observed by the detection of selected bacterial genera and species. Viable counts were determined by the plate count enumeration method with 10-fold dilutions. Fecal samples were stored in Wilkins-Chalgren anaerobic broth at −20 °C. Prior to analyses, 1 g of the feces was weighed and properly homogenized (vortex VELP Scientifica; Usmate, Italy) in 9 mL of physiological saline, serially diluted, and plated on Petri dishes. Each concentration gradient was measured in triplicate. The family *Enterobacteriaceae* and the genera *Clostridium and Lactobacillus,* as well as *E. coli* in fecal matter, were enumerated using the following selective cultivation media and conditions: for *Enterobacteriaceae*, VRBG agar (crystal-violet, neutral-red, bile, glucose agar; MILCOM, Tábor, Czech Republic) was used at 37 °C for 72 h; for *Clostridium*, RCM (Reinforced Clostridial Medium; MILCOM, Tábor, Czech Republic) was used at 37 °C for 72 h under anaerobic conditions after the previous inactivation of samples at 85 °C for 10 min; *E. coli* chromogenic TBX agar (tryptone, bile, X-glucuronide; MILCOM, Tábor, Czech Republic) was used at 37 °C for 72 h; and *Lactobacillus,* MRS 5.7 agar (de Man, Rogosa, and Sharpe; MILCOM, Tábor, Czech Republic) was used at 37 °C for 72 h under anaerobic conditions.

### 2.8. Statistical Analyses

For the data evaluation, Statgraphics^®^ Centurion XV (StatPoint, Inc., Warrenton, VA, USA) was used with two-way ANOVA for multiple comparisons, and the post hoc least significance difference test (LSD) was performed, considering the statistical significance at *p* < 0.05 (evaluation of the microbial counts).

Homoscedasticity and normality of data distribution were tested by the Levene and Shapiro-Wilk tests, respectively, as assumptions for the use of an analysis of variance (ANOVA). ANOVA followed by Tukey’s honestly significant difference (HSD) test was used for comparison of the data groups, and Dunnett’s test was used for comparison of the experimental groups with the control group. Statistica 13.1 software (StatSoft, Inc., Tulsa, OK, USA) and Real Statistics Resource Pack software (Release 7.2, Charles Zaiontz) were used for testing.

## 3. Results

### 3.1. Selenium Enrichment and Diets

Following lyophilization, viable counts (CFU) of the strains EF, ST, SeEF, and SeST were 10^9^ CFU/g. Both strains remained sufficiently viable, even after being processed into feed, and their counts reached approximately 10^4^ CFU/g in the final diet. Their presence in the live form confirms their desirable technological properties and ability to survive the heat treatment process.

Based on the contents of the selected biogenic elements, Table 2 shows the differences in the nutritional compositions of the experimental diets based on Altromin 1324 and Altromin C 1045. The selenium content in group A was determined by the addition of sodium selenite during the production of Altromin 1324 (producer information). Moreover, the addition of selenized LAB resulted in increased total Se contents (as expected) in groups D and F. The contents of the other elements remained unchanged in groups B–F after the addition of non-selenized/selenized LAB. The differences in contents of the elements between group A and groups B–F are shown in the diet matrix; maintenance diet Altromin 1324 is based on raw materials from cereal, while Se-deficient diet Altromin C 1045 is based on purified raw materials such as casein, starch, and sugars.

**Figure 1:**
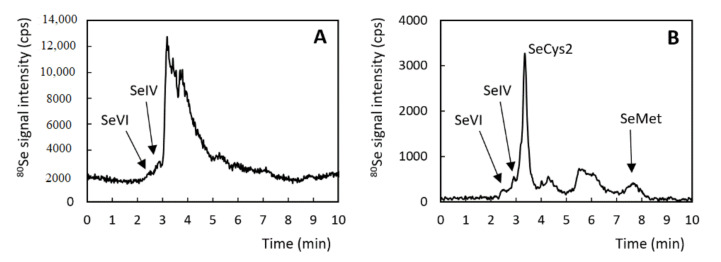
Chromatogram of the extracts of diet A (**A**) and diet B (**B**).

In addition to the total selenium content, Se speciation was analyzed in the feedstuff pellets (Figure 1). The efficiency of the enzymatic extraction was 40–85%. The speciation analyses revealed no significant differences in Se species abundances among groups B–F. The most abundant species was SeCys2, which accounted for 30–40% of total Se species. A smaller proportion was found for SeMet (8–16%), and minor species were represented by the inorganic Se forms, SeVI, and SeIV, with an average proportion of 2%. The rest of the selenium contents in the extract consisted of unidentified species. The chromatographic patterns obtained for the extract of diet A differed from each other. Although sodium selenite was added to this diet by the manufacturer, a higher proportion of inorganic selenium compounds was not found. It can thus be assumed that SeCys2 is a major selenium species in this diet; however, owing to the insufficient separation of the peaks in the chromatogram range of 3–5 min, this cannot be proven and evaluated.

**Figure 2:**
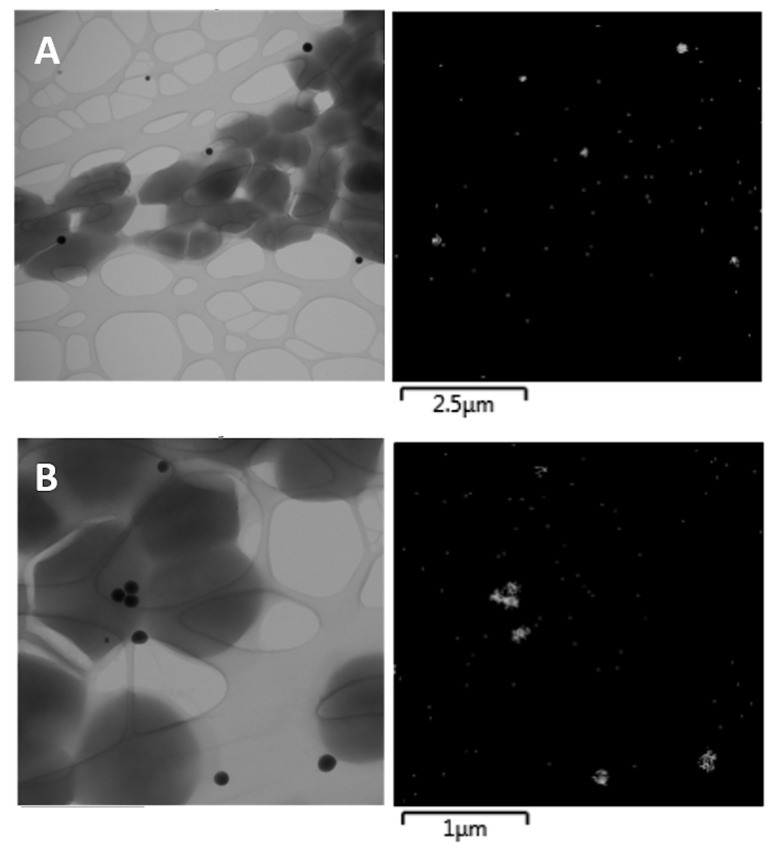
Bright field TEM images (**left**) of the CCDM 144 (**A**) and CCDM 922A (**B**) selenized strains with the corresponding energy-dispersive X-ray (EDX) maps of the signal intensity of selenium Se Kα_1_ spectral line (**right**).

Besides the selenium species mentioned above, selenium NPs were found in both selenized strains and characterized by TEM (Figure 2). TEM demonstrated the localization of the NPs outside of the cells. Se NPs inside the cells or cell membrane-bound NPs were not observed. The images showed that individual selenium NPs had approximately spherical shapes. The CCDM 144 strain produced NPs with a broad size distribution of 98–236 nm (average value of 171 nm), while the CCDM 922A strain produced NPs with sizes in the range of 42–185 nm (average value of 122 nm).

### 3.2. Effects of Selenium-Enriched Strains on Biochemical Parameters and Enzyme Activities in Tissue Homogenates and Blood

The diets had negligible effects on the body weight gains and weights of selected organs, except for lower brain weights in the Altromin 1324 group. In this group, the weights of the other organs also tended to be lower (Table 3).

Similarly, the serum concentrations of the total cholesterol, HDL cholesterol, and triacylglycerols (TAG) did not differ between the control and other groups (Table 4). The Altromin 1324 group tended to have higher lipid concentrations, which was consistent with the higher weight gain in this group.

Next, the activities (U/mL of the homogenate or serum) and concentrations (in ng/mL of the homogenate or serum) of the two antioxidant enzymes were measured. To gain more information on the oxidative stress status, the concentrations of MDA in the serum and heart were analyzed (Table 5). The effects of the diets were more pronounced in the heart, where a lower GR content was observed in all groups with added strains irrespective of Se fortification, with the activity of GR being higher in both diets with EF and SeEF (groups E and F). Notably, GPx was significantly less influenced by the diets. The MDA concentrations were also changed by both diets with EF/SeEF, especially in the heart, where the MDA concentrations were higher in the respective groups (E and F), unlike the changes in the serum (serum MDA was significantly lower only in group F, with SeEF).

### 3.3. Effects of Selenium-Enriched Strains on Total Selenium and Selenium-Species Contents in Tissue Homogenates

The concentrations of Se in the kidneys and liver of rats are higher than that in other organs [24]. Therefore, the total selenium content and selenium species in these tissues were analyzed. A higher selenium content (Table 6) was found in the kidneys of rats in groups A, D, and F, which were fed a Se-fortified diet. The contents of the other monitored elements were not affected by the diet, with the exception of the copper content, which was higher in group A. In the case of the liver samples (Table 7), the differences in the total Se contents between the groups with Se-fortified diets and non-selenized groups were significantly greater than those in the kidney samples. Moreover, the total Se content in group A was higher than those in groups D and F, where selenium was added in the form of selenized LAB. The contents of the other monitored elements were also not affected by the diet.

The aim of the speciation analysis was to determine whether the Se speciation varied with the Se and LAB contents in the diet. The efficiency of the enzymatic extraction of the selenium species from the kidney samples was 80 ± 6%. The HPLC column recovery for the kidney samples was 98% ± 8%, indicating that all selenium species in the extract were eluted from the column. The extraction efficiency of the selenium species from the liver samples was 74 ± 16%, and the column recovery was 103 ± 7%. These data indicate that a major part of the total Se was extracted, and no selenium species were captured on the HPLC column. The performed analysis thus provided a reliable overview of the distribution of selenium between the individual species.

**Figure 3:**
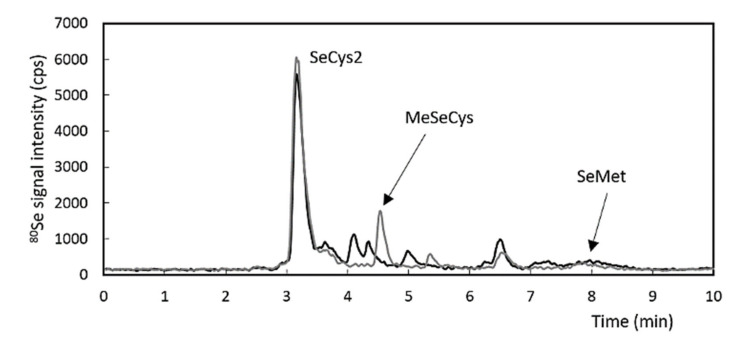
Chromatogram of the liver extract (black) and kidney extract (grey); both organ types were collected from group D rats (Altromin C 1045 + Se-enriched *Streptococcus thermophilus* (SeST)).

The speciation analysis revealed differences between the liver and kidney samples. SeCys2 was the major species and SeMet the minor species in both samples (Figure 3). The kidney samples also contained MeSeCys. The other minor Se species were unidentified. The species with retention times of 4.1, 4.3, and 5.0 min seemed to be specific for the liver, and the species with a retention time of 5.4 min were specific for the kidney samples. Unidentified species with a retention time of 6.6 min were observed in both sample types. No inorganic forms (SeVI or SeIV) were observed in the tissue samples.

**Figure 4:**
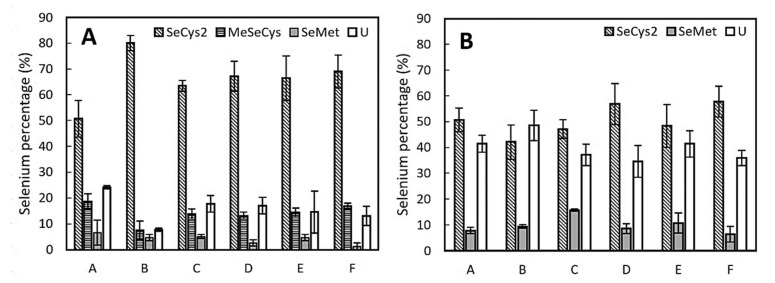
Selenium species representation expressed as a percentage of the total selenium content: (**A**) kidneys and (**B**) liver. The error bars indicate the standard deviation (*n* = 2). SeCys2 (selenocysteine), MeSeCys (Se-methylselenocysteine), SeMet (selenomethionine), and U (sum of unidentified species).

The average content of SeCys2 in the kidney samples (Figure 4) was the lowest in group A (51%) and the highest in control group B (80%). The SeCys2 percentages in the groups with added LAB in the diet (groups C–F) were similar (63–69%), regardless of their Se fortification. In the liver, as well as in the kidneys, SeCys2 was the most abundant species; however, the abundance in the liver (42–58%) was slightly lower than that in the kidneys. The SeCys2 percentages did not differ significantly for the individual groups, although higher average values were observed for groups D and F (57% and 58%, respectively), and the lowest average value was observed for control group B (42%). Besides SeCys2, SeMet was also found in the extracts. The proportions of SeMet were similar in all groups and reached values of 1–7% in the kidney samples and 6–16% in the liver samples. The last identified species, MeSeCys, was only found in the kidney extracts. The lowest content of MeSeCys (8%) was observed in control group B, while the other groups contained higher amounts (13–19%) of MeSeCys. Although the unidentified species constituted a minor portion (8–24%) of the total selenium content in the kidneys, a significant proportion (34–49%) was detected in the liver. All percentage values were based on an analysis of only two samples for each group. Due to the low number of analyzed samples, the actual variability or mean values may vary slightly.

### 3.4. Effect of Selenium-Enriched Strains on Bacterial Populations in Fecal Matter

Four different bacterial groups were subjected to analyses and were enumerated in the fecal matter collected from rat colons. The results are presented in Table 8. Compared with the group fed a standard maintenance diet (group A), the total counts (CFU/g) of *Clostridium* sp. decreased significantly (*p* ˂ 0.05) in all other experimental groups, whereas their reduction was even more apparent in the groups supplemented with the strain *Enterococcus faecium* (groups E and F). In group E, the counts of *Clostridium* sp. were reduced by more than two logarithmic orders. The *Enterobacteriaceae* and *E. coli* groups were slightly reduced in the supplemented groups (C, D, E, and F) and, also, in the Se-deficient group (B); however, this decrease was significant only in the group administered Se-enriched *Enterococcus faecium* (*p* ˂ 0.05), both in terms of *Enterobacteriaceae* and *E. coli*. In contrast, high viable counts of *Lactobacillus* sp. as a beneficial bacterial group were observed (ranging from 7.64 to 8.34 log CFU/g), which was a desirable count. The total counts of the *Lactobacillus* sp. were minimally affected among the experimental groups compared to the rats fed the standard maintenance diet (group A), although, in groups D and E, the viable counts were found to be slightly higher.

## 4. Discussion

In our previous study [22], we highlighted the abilities of *S. thermophilus* CCDM 144 and *E. faecium* CCDM 922A to accumulate up to 7.3 mg/g Se and 6.5 mg/g Se, respectively. Therefore, these strains were used for selenium enrichment of the SeST (group D) and SeEF (group F) diets in this study. The Se content in the diet of group A was derived from the natural Se contents in the ingredients of the diet and the added sodium selenite (according to the information provided by the producer). The diets were designed to obtain a difference of approximately 0.25-mg/kg Se between the Se-deficient diets (groups B, C, and E) and Se-enriched diets (groups A, D, and F). The speciation analysis revealed a different Se speciation in the diet of group A compared to the diets of the other groups. However, the differences in speciation may not represent the efficacies of the Se supplementation. Takahashi et al. [25] demonstrated an increased rapid absorption of SeMet and MeSeCys compared to the other Se species; however, at the same time, they also reported a lower efficiency of assimilation into selenoproteins. Moreover, although dietary Se amino acids are not directly incorporated into selenoproteins, all dietary forms of Se must be metabolized and enzymatically converted into selenide, which serves as a Se source for Se amino acids and for the subsequent protein synthesis [26]. The Se bioavailability in terms of selenoprotein production is thus similar for all Se species, with the main advantage of using organically bound Se to lower the toxicity. Se supplementation via LAB (groups D and F) is also supported by the fact that, in addition to organic Se species, LABs also contain nanoparticles that are considered to have a low toxicity [18].

In the presence of selenite, the bacterial cultivation medium was found to be red in color, which indicates the reduction of added selenite to elemental Se; this has been documented in previous studies as well [16,27]. Both strains used in this study produced spherical-shaped Se nanoparticles, as confirmed by the TEM results. *Streptococcus thermophilus* produced nanoparticles of sizes ranging from 98 to 236 nm, whereas *Enterococcus faecium* produced smaller nanoparticles with sizes ranging from 42 to 185 nm. In lactobacilli, a wider range of particle sizes, from 25 to 370 nm, was documented by Pescuma et al. [16]. Differences in particle sizes were also observed among the particles produced by different species of the same genus [28]. Moreover, differences were observed in terms of the location at which the NPs were produced. Although, in our case, Se NPs were located outside the cells, references to extracellular Se NPs are less common in the literature [29], and intracellular production is mostly described for LAB [27,30]. However, the presence of extracellular Se NPs could be advantageous over intracellular production because of their ease of accessibility and bioavailability. Moreover, Se NP formation is not a common feature of all LAB strains. Martínez et al. [31] demonstrated that Se NPs were produced in only eight (*Lactococcus lactis, Lactobacillus brevis* and *Lactobacillus plantarum, Fructobacillus tropaeola, Enterococcus casseliflavus*, and *Weissella cibaria*) out of 96 tested LAB strains. The binding of Se to the cell wall of LAB and their storage in the cell are mediated by complex processes that depend on factors such as the characteristics of the element (Se) and the specific physiological properties of individual LAB strains, as well as the cultivation media in which the bacteria are grown [19].

The LAB production of Se nanoparticles has been previously studied [16,28,32,33], and the NPs produced were found to be less toxic than other inorganic Se forms. The particle size depends on the strain used. In LABs, the sizes of the NPs produced ranged between 40 and 500 nm. According to Nagy et al. [28], smaller Se nanoparticles could be more toxic to the producing bacteria themselves. *Streptococcus thermophilus* tested in the aforementioned study produced Se nanoparticles of sizes ranging from 60 to 280 nm, which was followed by necrotic cell disruption. It has been reported that NPs are toxic to the cell, because they disrupt cell membranes. Therefore, the extracellular NPs produced by the two strains tested in this study could be advantageous, because they were not toxic to the cells themselves, in addition to their high bioavailability. Nevertheless, elemental Se is referred to as one of the least toxic Se forms with high bioavailability [18]. The absorption of Se NPs smaller than 100 nm in the gastrointestinal tract was proved to be 15–250 times higher than that of large-sized NPs [34]. Xu et al. [27] showed that *L. casei* with intracellularly accumulated Se NPs maintains an intestinal microbiota balance in response to oxidative stress and infection and exhibits an improved integrity of the intestinal epithelial barrier.

The antioxidant parameters were evaluated in the serum by measuring the GPx, GR enzyme activity, and MDA, a parameter of lipoperoxidation. In a recent study [5], a significant increase in GPx and SOD activities, along with reduced MDA concentrations and GSH activities, was observed in the serum of rats fed with Se- and Zn-enriched probiotics. Accordingly, the beneficial effects of Se-enriched strains improving the antioxidant status in comparison with the control group were observed in this study as well. The MDA values provide a rough estimate of the oxidative stress in the tissue. We observed lower concentrations of serum MDA following Se supplementation, as in the study of Malyar et al. [5], which was accompanied by higher serum activities of antioxidative enzymes. This association, however, is not supported by the results of our study, as the serum and heart GPx activities did not change between the observed groups. The activity of GPx was lower in the rat heart and brain tissues than that in the other tissues. These tissues differed by both concentrations of Se (app. three-fold higher in the heart) and the relative amount of Se bound to GPx (20% in the heart vs. 3% in the brain) [35]. In another study, the supplementation with SeMet revealed no effect on the GPX heart activity in rodents [36]. Following Se supplementation, we observed the changes in the heart GR activity. This enzyme is not Se-dependent but reduces glutathione to maintain a reduced glutathione pool [37].

In the rat hearts of all the groups in which LAB strains were administered, changes in the oxidative stress parameters were observed. In heart tissues of the groups with added ST and SeST strains (groups C and D), the concentration of MDA only tended to increase; therefore, lower amounts of GR (reduced glutathione-forming enzyme), together with its sustained activity, were most likely sufficient to maintain an acceptable level of oxidative stress. In contrast, the higher indices of oxidative stress in the heart following the diets with EF and SeEF (groups E and F)—observed as high heart MDA concentrations—were associated with the increased activity of GR despite its lower amounts. We can speculate that it may be a result of the efforts to maintain a reduced pool of GSH capable of inactivating elevated lipoperoxide levels, which is reflected in the increased MDA concentrations in the heart tissue of these rats. At the moment, we can only hypothesize why this happened in just the EF and SeEF groups. The effect of the strain *Enterococcus faecium* CCDM 922A remains speculative itself, since it has been reported to interfere with the lipid metabolism and exhibits the capacity to reduce low-density lipoprotein and very low-density lipoprotein cholesterol in rats [23]. The two diets, standard Altromin 1234 and Se-deficient Altromin C 1045, differed both in vitamin E content (75 mg/kg vs. 150 mg/kg) and Se concentration (408 µg/kg vs. 108 µg/kg). The parameters of the antioxidative system did not differ between these two groups; thus, no effect or sum of opposite effects can be expected. In rats, Se and vitamin E exhibited dietary compensatory relationships and were found to act synergistically [38,39]. Moreover, various additional interactions between Se and other trace elements or nutrients have been described [40]. The content of Cu, Fe, Cr, and Zn in the liver and kidneys did not differ in the EF/SeEF groups compared to the B–D groups, and for this reason, the influence of other elements may be ruled out. To consider all the possible parameters, we also can speculate the stress response to hunger/conditions before decapitation, given that groups E and F were euthanized last. It is known that different selenoproteins are involved in the cardiovascular stress response; among them, the GPx family is one of the best characterized. Nevertheless, the exact role of the other selenoproteins involved (e.g., thioredoxin reductase, thyroid hormone deiodinases, selenoprotein R, and selenoprotein K) remains only partly understood, and further investigation of the Se-dependent effects at the molecular level is needed [41].

The Se levels were monitored in the liver and kidney samples, as these organs are involved in Se regulation, the production of excretory selenium forms, and generally accumulate a higher amount of selenium in comparison to other organs [42]. The total observed selenium levels in the liver (0.14–0.38 mg/kg) and kidneys (0.8–1.2 mg/kg) agreed with those observed in other studies focusing on animals fed with Se-enriched foodstuffs [38,43,44,45]. In these studies, the Se contents in the liver samples were found to be in the range of 0.25–5.0 mg/kg, while those in the kidney samples were in the range of 1.0–6.7 mg/kg.

Higher Se levels in Se-enriched diets resulted in increased Se contents in the kidneys and liver. Although the Se contents in the kidney samples of the groups fed with Se-enriched diets were similar, and the differences were not significant (*p* < 0.05) in all cases, the highest average Se content was observed in the kidney samples from group A, the diets of which contained both inorganic and organic Se. The highest Se content in group A was also observed in the liver samples; this value was significantly higher than the Se content in groups D and F with diets containing organically bound Se only. However, the knowledge on whether the addition of inorganic Se into diet leads to higher Se contents in rat liver and kidneys compared to the addition of organic forms is sporadic in the literature. Rýdlová et al. [38] confirmed that the intake of Se-enriched defatted rapeseed (0.2 mg/kg Se) added had no effect on the Se content in the liver and kidneys of Wistar and spontaneously hypertensive rats in comparison to the control group. The opposite trend has been described more often; Zhou et al. [46] highlighted that Se accumulation from organic Se supplements was higher than that from inorganic supplements in most biological tissues of Sprague–Dawley rats. The Se content in the liver of rats fed a diet fortified with Se-enriched *Bifidobacterium longum* or selenized yeast was much higher than that of rats fed a selenite-enriched diet. The Se content in the kidneys showed the same trend, but the differences were smaller for all three groups. Similarly, Zhang et al. [47] found higher Se levels in the kidneys and liver of rats administered a diet fortified with Se-enriched garlic and a mixture of non-selenized garlic and selenite than in the kidneys and liver of rats administered a selenite-enriched diet only. Qin et al. [43] observed a higher Se content in the kidneys and liver of lambs fed Se-enriched yeast and Se-enriched probiotics (a mixture of *Lactobacillus* and *Saccharomyces cerevisiae*) than in the kidneys and liver of lambs fed a selenite-fortified diet. Marounek et al. [48] observed the same effect in rabbits fed Se-enriched yeast, Se-enriched algae, and selenite-fortified diets. Some studies also showed no difference between the organic and inorganic Se sources in terms of their accumulation in organs. Sobeková et al. [45] confirmed in the case of lambs that an increased dietary Se intake in the form of selenite and Se-enriched yeast led to significantly higher Se contents in the liver and kidneys in comparison to the control; however, no significant difference between diets enriched with selenite and Se-enriched yeast was observed. These findings were supported by Han et al. [44], who fed hens a Se-fortified diet (selenite and Se-enriched yeasts).

Due to the importance of the liver and kidneys in the metabolism of Se, many studies also focused on Se speciation in these organs. SeCys2 or SeCys are described as the major Se species in the kidneys and liver, followed by SeMet as the second-most abundant species for various animal species, e.g., broiler chicks [49], rain trout [50], rats [51], or lambs [52]. However, in some animals—for example, sheep [42] and lambs [53]—similar percentages of SeMet, SeCys2, and MeSeCys were observed. SeCys2 thus appears to be the dominant species in the liver and kidneys of most animals, although, in some cases, SeMet and MeSeCys may be on par with SeCys2. Our results were consistent with the published data. SeCys2 was found to be the major Se species in the liver samples (42–58%) and kidney samples (51–80%). Instead of SeCys2, only SeMet was identified in the liver, and its percentage ranged from 6% to 16%. In the kidneys, although SeMet was only found in trace amounts (1–7%), MeSeCys was present in larger amounts (8–19%). Other species in the liver and kidneys were unidentified. A statistically significant difference (*p* < 0.05) was observed only in the proportion of SeCys2 and MeSeCys in the kidneys between groups A and B, but the differences were not significant. Thus, the addition of Se to the diet, whether in its organic or inorganic form, did not alter the Se speciation. Similar results were obtained by Juniper et al. [52] in the case of lambs fed a Se-enriched yeast diet. The kidney and liver samples were comprised of a large proportion of the total Se as SeCys, and no change in speciation was observed in comparison to the control group. Small changes in Se speciation were observed in the study of Zhao et al. [49], who fed broiler chicks with the inorganic and organic forms of Se. SeCys2 and SeMet were found to be the major and minor species in the livers of chicks, respectively. In the case of Se-enriched yeast diets, a larger proportion of SeMet (3.5–7.3-fold) was observed in comparison to the diets containing selenite.

The composition of the intestinal microbiota can be affected by numerous factors that alter the microbial homeostasis. These factors include stress, dietary changes, the overuse of antibiotics, and gastrointestinal or other infections. The intestinal redox status and inflammatory levels are also strongly associated with alterations in the gut microbiota [54]. The Se status has been reported as a regulator of intestinal microbiota, especially when the Se availability is limited [55]. Individual bacterial species and strains are differently sensitive to Se concentrations; a concentration of Se that is tolerable by one strain may be toxic to another. The tolerance of strains to Se can be attributed to the differences in their individual capabilities to uptake, store, use, and remove Se from cells [56]. Recent studies have supported the theory that the gut microbiota also plays a role in Se metabolism in the host by metabolizing bioselenocompounds [57], whereas both organic and inorganic Se have been proven to have an effect on the community structure of the gut microbiota [54]. Additionally, a Se-deficient diet has been associated with damaged intestinal barrier function and imbalanced gut microbiota [54].

Following feedstuff processing, sufficient counts of viable cells (approximately 10^4^ CFU/g) of LAB were observed in the pellets of the experimental diet. Due to the technological properties of the strains CCDM 144 and CCDM 922A and their survival during processing, they are widely employed in industrial applications. The presence of live bacteria by itself provides benefits—for example, in the production of acids, bacteriocins, and other antimicrobial compounds and the competitive exclusion of pathogens that are well-known in LAB strains [12]. Provided that the LABs are additionally enriched with Se, Zn, or other essential minerals, the delivery of these nutrients can also positively affect the gut microbiota and, hence, multiply the overall beneficial effect of the LAB or probiotic LAB administration. Dietary Se supplementation, whether in organic or inorganic forms via Se-enriched LAB or yeasts, was associated with modulation of the gut microbiota, as demonstrated in several previous studies [56,58,59,60]. For example, Se nanoparticles have been documented to increase the abundance of *Lactobacillus* sp. or *Faecalibacterium prausnitzii* and reduce the abundances of harmful strains—for example *Enterococcus cecorum* in poultry [61,62]. *Lactobacillus* sp. is an important LAB species, which includes many important strains with probiotic functions contributing to the maintenance of healthy and balanced gut ecosystems. In this study, we observed desirable high levels of *Lactobacillus* sp. (10^7^–10^8^ CFU/g) in the fecal matter of rats in all experimental groups, regardless of the final Se concentration in the diet and regardless of supplementation with LAB or Se-enriched LAB. In contrast, the highest influence of the supplementation was observed in the *Clostridium* sp. The highest counts of *Clostridium* sp. were detected in group A, which was administered a standard maintenance diet, whereas lower counts were observed in the remaining groups—that is, groups administered both Se-enriched LAB and LAB without Se-enrichment, as well as in groups administered the Se-deficient diet (groups B–F). Consistent with our results, *Clostridiaceae* were also more enhanced in the group of normal diet-fed rats compared to the low-Se diet-fed group in the study by Cheng et al. [54]. Notably, with the exception of the group fed a standard diet (group A), in all other groups, the levels of *Clostridium* sp. diminished, even in the groups (D and F) in which Se-enriched LAB were supplemented; thus, the final Se contents in the groups fed these two diets were comparable with those in the groups fed a standard diet (group A). We can therefore assume that the growth decrease is most probably not caused by Se itself. Among the other elements, the Fe content was more than three-fold higher in the group administered the standard Altromin 1324-based diet (group A) in comparison with that in the group administered the Altromin C1045-based diet (groups B-F). This is an important fact, because iron is known to play an important role in bacteria, especially in clostridial metabolism. Iron is involved in the active metal sites of enzymes or proteins such as ferredoxin, hydrogenase, and cytochrome, as well as participates in microbial energy conversion [63]. The *Clostridium* sp. belongs to fermentative-type iron reducers with a robust dissimilatory iron reduction capacity, efficiently stimulating cell growth and enhancing nutrient uptake [63,64]. This could explain the enhanced *Clostridium* counts in the group administered with the standard diet (group A), where the clostridia had higher amounts of available iron. The genus *Clostridium* includes species such as *C. butyricum* and *C. perfringens*, which are often associated with the development of necrotizing enterocolitis [65]. In contrast, nonpathogenic butyrate-producing strains belong to this group of bacteria [66]. Other bacterial groups analyzed in the fecal matter were the family *Enterobacteriaceae* and, within it, the *E. coli* group as a representative fecal coliform bacterium present in the colon of humans and warm-blooded animals. Only in group F, where the Se-enriched *Enterococcus faecium* strain was supplemented, the total counts of both *Enterobacteriaceae* and *E. coli* decreased significantly. In a canine model [12], following the oral administration of selenium/zinc-enriched probiotics, similar to our results, a decrease of *E. coli* in the feces was reported, along with an increase in *Lactobacillus* sp. and *Bifidobacterium* sp.

The gut microbiota may also utilize Se for the expression of its own selenoproteins. In the well-described yeast *Saccharomyces cerevisiae* model, the addition of inorganic Se into the cultivation media led to the production of SeMet [67]. SeMet and MeSeCys, two of the most common organic Se forms, were shown to be absorbed more efficiently than other selenocompounds [25]. Thus, the gut microbiota plays a role in Se metabolism. The gut microbiota form a complex ecosystem and multispecies environment where many different relationships, including commensalism, mutualism, or cross-feeding, exist between the resident gut microbiotas. Microbiota self-metabolizes Se and, thus, by Se supplementation, improves its own state; concomitantly, it helps to make dietary Se more available to the host organism.

This is the first report on the supplementation of selenized forms of the commercially used strains *Enterococcus faecium* CCDM 922A and *Streptococcus thermophilus* CCDM 144 in an in vivo model and the effect of their supplementation on the biological functions of rats. The limitations of the study included missing more extensive speciation patterns and mineral contents in other tissues, such as brain/heart tissues, which may shed more light into the findings in heart tissues. As Se is known to accumulate primarily in the liver and kidney tissues, other organs were not subjected to the Se content analysis, nor to the determination of the Se species in these tissues. Additionally, due to numerous parameters having an affinity towards oxidative stress, such as superoxide dismutase, catalase, oxidized/reduced glutathione ratio, isoprostanes, and others, we were not able to thoroughly analyze all of them and, thus, give a more definitive picture of the influence of all the possible biomarkers involved. Overall, the existing in vivo studies supplementing Se in the form of Se-enriched LAB are hardly comparable due to variables such as the different supplementation strategies, possible effects of other nutrients, vitamins and antioxidants, differences in the administered Se forms, etc.

## 5. Conclusions

Selenized forms of the strains *Streptococcus thermophilus* CCDM 144 and *Enterococcus faecium* CCDM 922A were proven to represent a source of organically bound Se, SeCys2 being the most abundant Se species, followed by SeMet. These species were also found in the liver and kidney tissues of rats. In addition, MeSeCys was also detected but only in the kidneys. Moreover, the production of extracellular Se NPs of different sizes was confirmed by TEM EDX in both the strains. We showed that the administration of Se-enriched LAB caused no significant changes in the basic metabolic characteristics of the experimental animals. Rats fed a standard maintenance diet and those fed the experimental diet fortified with the Se-enriched LAB diet showed similar total Se concentrations and Se speciation patterns in both the kidneys and livers. Furthermore, in the Se-enriched LAB tested in this study, no macroscopic effects on the liver, kidneys, heart, and brain were observed, with no apparent influence on the basic parameters of the lipid metabolism. The antioxidative system capacity was altered only in the heart tissue of rats fed the experimental diet. Together, both the strains—*Streptococcus thermophilus* CCDM 144 and *Enterococcus faecium* CCDM 922A—may have the potential for the production of Se-enriched LAB, representing a source of organic forms of Se and Se NPs. No adverse effects on the biological functions of the rats were observed. Our findings provide a foundation to further develop and establish their safety for industrial application in foods and food supplements.

## Figures and Tables

**Table 1:** Selenium concentrations and its forms in the experimental groups; data are presented as the mean ± expanded uncertainty with coverage factor *k* = 2.

	Se Concentration (mg/kg)	Added Se Form
A—Altromin 1324	0.408 ± 0.086	inorganic
B—Altromin C 1045	0.183 ± 0.039	-
C—Altromin C 1045 + ST	0.233 ± 0.076	-
D—Altromin C 1045 + SeST	0.439 ± 0.016	Organically bound
E—Altromin C 1045 + EF	0.185 ± 0.032	-
F—Altromin C 1045 + SeEF	0.445 ± 0.078	Organically bound

ST, *Streptococcus thermophilus*, SeST, selenium-enriched *Streptococcus thermophilus*, EF, *Enterococcus faecium*, and SeEF, selenium-enriched *Enterococcus faecium*.

**Table 2:** Total contents of the elements in the feedstuff (mg/kg wet weight, *n* = 3). The averages marked by the same letter do not significantly differ at *p* < 0.05 within the individual columns; data are presented as the mean ± expanded uncertainty with a coverage factor *k* = 2.

Sample	Cu (mg/kg)	Zn (mg/kg)	Cr (mg/kg)	Fe (mg/kg)
A—Altromin 1324	26.0 ± 9.0 ^a^	63.7 ± 9.9 ^a^	51.4 ± 8.4 ^a^	418 ± 43 ^a^
B—Altromin C 1045	14.4 ± 5.4 ^b^	48.7 ± 2.3 ^b^	37.8 ± 2.9 ^b^	125 ± 11 ^b^
C—Altromin C 1045 + ST	13.8 ± 4.8 ^b^	47.7 ± 1.2 ^b^	37.3 ± 6.4 ^b^	123 ± 28 ^b^
D—Altromin C 1045 + SeST	13.2 ± 2.3 ^b^	47.8 ± 6.8 ^b^	37.4 ± 5.6 ^b^	117 ± 16 ^b^
E—Altromin C 1045 + EF	13.3 ± 3.3 ^b^	46.8 ± 3.9 ^b^	38.2 ± 7.4 ^b^	119 ± 13 ^b^
F—Altromin C 1045 + SeEF	15.8 ± 5.5 ^b^	47.1 ± 2.0 ^b^	36.4 ± 6.8 ^b^	122 ± 11 ^b^

**Table 3:** The body weights and weights of organs in grams (*n* = 8). Data are presented as the mean (standard deviation). ^a,b^–values in columns with different supercase letters significantly differ (*p* < 0.05).

Group	Body Weight Gain (g)	Heart (g)	Brain (g)	Liver (g)	Kidneys (g)
A—Altromin 1324	102 (60) ^a^	1.04 (0.13) ^a^	1.58 (0.20) ^b^	13.7 (4.8) ^a^	2.86 (0.46) ^a^
B—Altromin C 1045	85 (41) ^a^	1.25 (0.11) ^a^	2.12 (0.08) ^a^	14.3 (2.7) ^a^	3.27 (0.34) ^a^
C—Altromin C 1045 + ST	89 (67) ^a^	1.34 (0.25) ^a^	2.15 (0.10) ^a^	14.8 (2.7) ^a^	3.38 (0.29) ^a^
D—Altromin C 1045 + SeST	68 (35) ^a^	1.27 (0.15) ^a^	2.14 (0.06) ^a^	14.3 (1.4) ^a^	3.32 (0.24) ^a^
E—Altromin C 1045 + EF	75 (42) ^a^	1.25(0.14) ^a^	2.13 (0.06) ^a^	15.1 (1.8) ^a^	3.40 (0.46) ^a^
F—Altromin C 1045 + SeEF	65 (50) ^a^	1.21 (0.11) ^a^	2.09 (0.13) ^a^	14.6 (2.0) ^a^	3.30 (0.32) ^a^

**Table 4:** Serum concentrations of the basic lipid parameters (mmol/L, *n* = 8). Data are presented as the mean (standard deviation). ^a^–values in columns with the same superscript letter do not significantly differ (*p* < 0.05).

Group	Cholesterol mmol/L	TAG (mmol/L)	HDL Cholesterol (mmol/L)
A—Altromin 1324	1.69 (0.23) ^a^	1.01 (0.44) ^a^	1.25 (0.26) ^a^
B—Altromin C 1045	1.55 (0.25) ^a^	0.79 (0.45) ^a^	1.16 (0.22) ^a^
C—Altromin C 1045 + ST	1.46 (0.22) ^a^	0.49 (0.38) ^a^	1.04 (0.12) ^a^
D—Altromin C 1045 + SeST	1.44 (0.18) ^a^	0.59 (0.49) ^a^	1.03 (0.14) ^a^
E—Altromin C 1045 + EF	1.49 (0.27) ^a^	0.87 (0.43) ^a^	1.09 (0.23) ^a^
F—Altromin C 1045 + SeEF	1.53 (0.24) ^a^	0.66 (0.38) ^a^	1.10 (0.19) ^a^

TAG–triacylglycerols; HDL–high-density lipoprotein cholesterol.

**Table:** (**A**)

Group	Heart GPx (ng/mL)	Heart GPx (U/mL)	Brain GPx (ng/mL)	Brain GPx (U/mL)	Serum GPx (ng/mL)	Serum GPx (U/mL)
A—Altromin 1324	45.7 (8.7) ^a^	13.3 (3.7) ^a^	51.8 (7.0) ^a^	15.3 (2.3) ^a^	27.9 (2.09) ^b^	0.34 (0.39) ^a^
B—Altromin C 1045	41.9 (4.1) ^a^	14.6 (1.3) ^a^	49.8 (5.3) ^a^	14.5 (3.3) ^a^	25.1 (2.58) ^a^	1.49 (2.27) ^a^
C—Altromin C 1045 + ST	28.6 (12.0) ^b^	14.8 (2.3) ^a^	49.8 (4.6) ^a^	14.7 (4.2) ^a^	24.6 (1.84) ^a^	0.15 (0.09) ^a^
D—Altromin C 1045 + SeST	34.7 (8.3) ^a^	12.1 (6.1) ^a^	50.9 (3.3) ^a^	12.0 (2.7) ^a^	25.0 (1.90) ^a^	0.12 (0.07) ^a^
E—Altromin C 1045 + EF	39.3 (5.7) ^a^	15.7 (2.2) ^a^	50.1 (4.5) ^a^	11.3 (1.4) ^a^	25.5 (1.16) ^a^	0.90 (1.63) ^a^
F—Altromin C 1045 + SeEF	40.4 (6.1) ^a^	14.5 (2.7) ^a^	51.8 (1.8) ^a^	9.4 (1.3) ^b^	24.2 (1.69) ^a^	0.16 (0.29) ^a^

**Table:** (**B**)

Group	Heart GR (ng/mL)	Heart GR (U/mL)	Brain GR (ng/mL)	Brain GR (U/mL)	Serum MDA (ng/mL)	Brain MDA (ng/mL)	Heart MDA (ng/mL)
A—Altromin 1324	88.8 (11.9) ^a^	138.8 (5.0) ^a^	0.67 (0.46) ^a^	158.9 (23.5) ^a^	280 (97) ^a^	469 (86) ^a^	348 (89) ^a^
B—Altromin C 1045	75.5 (10.9) ^a^	135.7 (6.5) ^a^	0.35 (0.23) ^a^	178.1 (67.1) ^a^	305 (182) ^a^	454 (49) ^a^	288 (144) ^a^
C—Altromin C 1045 + ST	43.9 (15.2) ^b^	138.6 (12.2) ^a^	0.48 (0.23) ^a^	156.8 (8.3) ^a^	296 (35) ^a^	414 (63) ^a^	348 (61) ^a^
D—Altromin C 1045 + SeST	43.2 (7.9) ^b^	136.0 (13.7) ^a^	0.60 (0.13) ^a^	150.0 (10.1) ^a^	260 (81) ^a^	432 (48) ^a^	358 (92) ^a^
E—Altromin C 1045 + EF	49.2 (14.8) ^b^	149.4 (7.6) ^b^	0.31 (0.13) ^a^	144.4 (18.8) ^a^	201 (59) ^a^	445 (39) ^a^	570 (100) ^b^
F—Altromin C 1045 + SeEF	38.3 (10.1) ^b^	155.3 (3.9) ^b^	0.38 (0.28) ^a^	139.0 (8.0) ^a^	164 (51) ^b^	485 (79) ^a^	460 (65) ^b^

GPx–glutathione peroxidase; GR–glutathione reductase; MDA–malondialdehyde.

**Table 6:** Total contents of the elements in rat kidneys (mg/kg wet weight, *n* = 8). The averages marked by the same letter do not significantly differ at *p* < 0.05 within the individual columns; data are presented as the mean (standard deviation).

Sample	Se (mg/kg)	Cu (mg/kg)	Zn (mg/kg)	Cr (mg/kg)	Fe (mg/kg)
A—Altromin 1324	1.22 (0.17) ^a^	12.0 (3.4) ^a^	16.6 (2.9) ^a^	0.233 (0.039) ^a^	88 (27) ^a^
B—Altromin C 1045	0.79 (0.15) ^b^	6.2 (1.5) ^b^	17.3 (2.7) ^a^	0.254 (0.045) ^a^	131 (69) ^a^
C—Altromin C 1045 + ST	0.79 (0.07) ^b^	6.4 (1.4) ^b^	17.4 (1.8) ^a^	0.205 (0.046) ^a^	132 (84) ^a^
D—Altromin C 1045 + SeST	1.08 (0.18) ^ac^	7.4 (2.9) ^b^	17.5 (1.4) ^a^	0.221 (0.026) ^a^	94 (47) ^a^
E—Altromin C 1045 + EF	0.81 (0.16) ^b^	6.7 (2.2) ^b^	17.2 (2.1) ^a^	0.243 (0.076) ^a^	90 (54) ^a^
F—Altromin C 1045 + SeEF	0.89 (0.12) ^bc^	5.0 (0.7) ^b^	16.1 (2.4) ^a^	0.200 (0.027) ^a^	77 (38) ^a^

**Table 7:** Total contents of the elements in rat livers (mg/kg wet weight, *n* = 8). The averages marked by the same letter do not significantly differ at *p* < 0.05 within the individual columns; data are presented as the mean (standard deviation).

Sample	Se (mg/kg)	Cu (mg/kg)	Zn (mg/kg)	Cr (mg/kg)	Fe (mg/kg)
A—Altromin 1324	0.383 (0.058) ^a^	2.25 (0.26) ^a^	11.9 (1.7) ^a^	0.070 (0.017) ^a^	131 (37) ^a^
B—Altromin C 1045	0.137 (0.018) ^b^	2.43 (0.25) ^a^	11.9 (1.5) ^a^	0.067 (0.011) ^a^	113 (17) ^ab^
C—Altromin C 1045 + ST	0.152 (0.023) ^b^	2.42 (0.27) ^a^	11.5 (1.1) ^a^	0.074 (0.015) ^a^	119 (13) ^ab^
D—Altromin C 1045 + SeST	0.252 (0.055) ^c^	2.37 (0.31) ^a^	11.2 (2.1) ^a^	0.072 (0.015) ^a^	110 (14) ^ab^
E—Altromin C 1045 + EF	0.142 (0.029) ^b^	2.44 (0.21) ^a^	10.5 (1.1) ^a^	0.066 (0.016) ^a^	104 (28) ^ab^
F—Altromin C 1045 + SeEF	0.213 (0.039) ^c^	2.31 (0.12) ^a^	11.1 (1.3) ^a^	0.072 (0.013) ^a^	105 (23) ^b^

**Table 8:** Effects of the experimental diets on the intestinal bacterial groups.

Groups	Total Counts (log CFU/g)			
	*Clostridium*	*Enterobacteriaceae*	*Escherichia coli*	*Lactobacillus*
A—Altromin 1324	3.04 (0.46) ^d^	5.01 (0.67) ^b^	4.98 (0.72) ^b^	7.78 (0.64) ^ab^
B—Altromin C 1045	1.99 (0.43) ^c^	4.42 (0.57) ^b^	4.37 (0.62) ^b^	7.87 (0.62) ^ab^
C—Altromin C 1045 + ST	1.85 (0.60) ^bc^	4.74 (0.51) ^b^	4.58 (0.42) ^b^	7.98 (0.48) ^ab^
D—Altromin C 1045 + SeST	1.51 (0.80) ^bc^	4.79 (0.77) ^b^	4.73 (0.64) ^b^	8.29 (0.61) ^b^
E—Altromin C 1045 + EF	0.96 (0.32) ^a^	4.68 (0.92) ^b^	4.40 (0.53) ^b^	8.34 (0.55) ^b^
F—Altromin C 1045 + SeEF	1.39 (0.38) ^ab^	3.39 (0.77) ^a^	3.25 (1.04) ^a^	7.64 (0.61) ^a^

ST, *Streptococcus thermophilus*, SeST, selenium-enriched *Streptococcus thermophilus*, EF, *Enterococcus faecium,* and SeEF, selenium-enriched *Enterococcus faecium*. Data are presented as the mean (standard deviation) from triplicate determination. Values within columns with different superscripts indicate a significant difference at *p* < 0.05. CFU: colony-forming units.

## Data Availability

All data are presented in the paper.

## References

[1] WHO (2015). Cardiovascular Diseases (CVDs): Fact. Sheet N 317.

[2] Chua K.J., Kwok W.C., Aggarwal N., Sun T., Chang M.W. (2017). Designer Probiotics for the Prevention and Treatment of Human diseases. Curr. Opin. Chem. Biol..

[3] Amaretti A., Di Nunzio M., Pompei A., Raimondi S., Rossi M., Bordoni A. (2013). Antioxidant Properties of Potentially Probiotic Bacteria: In Vitro and in Vivo Activities. Appl. Microbiol. Biotechnol..

[4] Steinbrenner H., Speckmann B., Klotz L.O. (2016). Selenoproteins: Antioxidant Selenoenzymes and Beyond. Arch. Biochem. Biophys..

[5] Malyar R.M., Li H., Liu D., Abdulrahim Y., Farid R.A., Gan F., Ali W., Enayatullah H., Banuree S.A.H., Huang K. (2020). Selenium/Zinc-Enriched Probiotics Improve Serum Enzyme Activity, Antioxidant Ability, Inflammatory Factors and Related Gene Expression of Wistar Rats Inflated Under Heat Stress. Life Sci..

[6] Samarghandian S., Farkhondeh T., Samini F., Borji A. (2016). Protective Effects of Carvacrol Against Oxidative Stress Induced by Chronic Stress in Rat‘S Brain, Liver and Kidney. Biochem. Res. Int..

[7] Brigelius-Flohé R. (2018). Selenium in Human Health and Disease: An Overview. Mol. Integr. Toxicol..

[8] Rayman M.P. (2012). Selenium and Human Health. Lancet.

[9] Yi H.W., Zhu X.X., Huang X.L., Lai Y.Z., Tang Y. (2020). Selenium-Enriched Bifidobacterium Longum Protected Alcohol and High Fat Diet Induced Hepatic Injury in Mice. Chin. J. Nat. Med..

[10] Sun Z., Xu Z., Wang D., Yao H., Li S. (2018). Selenium Deficiency Inhibits Differentiation and Immune Function and Imbalances the Th_1_/Th_2_ of Dendritic Cells. Metallomics.

[11] Jamilian M., Mansury S., Bahmani F., Heidar Z., Amirani E., Asemi Z. (2018). The Effects of Probiotic and Selenium Co-Supplementation on Parameters of Mental Health, Hormonal Profiles, and Biomarkers of Inflammation and Oxidative Stress in Women with Polycystic Ovary Syndrome. J. Ovarian Res..

[12] Ren Z., Zhao Z., Wang Y., Huang K. (2011). Preparation of Selenium/Zinc-Enriched Probiotics and Their Effect on Blood Selenium and Zinc Concentrations, Antioxidant Capacities, and Intestinal Microflora in Canine. Biol. Trace Elem. Res..

[13] Yang J., Huang K., Qin S., Wu X., Zhao Z., Chen F. (2009). Antibacterial Action of Selenium-Enriched Probiotics against Pathogenic *Escherichia coli*. Dig. Dis. Sci..

[14] Oraby M.M., Allababidy T., Ramadan E.M. (2015). The Bioavailability of Selenium in *Saccharomyces cerevisiae*. Ann. Agric. Sci..

[15] Lamberti C., Mangiapane E., Pessione A., Mazzoli R., Giunta C., Pessione E. (2011). Proteomic Characterization of a Selenium-Metabolizing Probiotic *Lact. Reuteri* Lb2 BM for Nutraceutical Applications. Proteomics.

[16] Pescuma M., Gomez-Gomez B., Perez-Corona T., Font G., Madrid Y., Mozzi F. (2017). Food Prospects of Selenium Enriched-*Lactobacillus acidophilus* CRL 636 and *Lactobacillus reuteri* CRL 1101. J. Funct. Foods.

[17] Pusztahelyi T., Kovács S., Pócsi I., Prokisch J. (2015). Selenite-Stress Selected Mutant Strains of Probiotic Bacteria for Se Source Production. J. Trace Elem. Med. Biol..

[18] Nagy G., Benko I., Kiraly G., Voros O., Tanczos B., Sztrik A., Takács T., Pocsi I., Prokisch J., Banfalvi G. (2015). Cellular and Nephrotoxicity of Selenium Species. J. Trace Elem. Med. Biol..

[19] Mrvčić J., Stanzer D., Solić E., Stehlik-Tomas V. (2012). Interaction of Lactic Acid Bacteria with Metal Ions: Opportunities for Improving Food Safety and Quality. World J. Microbiol. Biotechnol..

[20] Rother M., Hatfield D., Berry M., Gladyshev V. (2011). Selenium Metabolism in Prokaryotes. Selenium.

[21] Zhang B., Zhou K., Zhang J., Chen Q., Liu G., Shang N., Qin W., Li P., Lin F. (2009). Accumulation and Species Distribution of Selenium in Se-Enriched Bacterial Cells of the *Bifidobacterium animalis* 01. Food Chem..

[22] Krausova G., Kana A., Hyrslova I., Mrvikova I., Kavkova M. (2020). Development of Selenized Lactic Acid Bacteria and Their Selenium Bioaccummulation Capacity. Fermentation.

[23] Hyrslova I., Krausova G., Bartova J., Kolesar L., Jaglic Z., Stankova B., Curda L. (2016). Characterization of *Enterococcus Faecium* CCDM 922 in Respect of its Technological and Probiotic Properties. Int. J. Curr. Microbiol. Appl. Sci..

[24] Shiobara Y., Ogra Y., Suzuki K.T. (2000). Exchange of Endogenous Selenium for Dietary Selenium as 82 Se-Enriched Selenite in Brain, Liver, Kidneys and Testes. Life Sci..

[25] Takahashi K., Suzuki N., Ogra Y. (2017). Bioavailability Comparison of Nine Bioselenocompounds In Vitro and In Vivo. Int. J. Mol. Sci..

[26] Shini S., Sultan A., Bryden W.L. (2015). Selenium Biochemistry and Bioavailability: Implications for Animal Agriculture. Agriculture.

[27] Xu C., Guo Y., Qiao L., Ma L., Cheng Y., Roman A. (2018). Biogenic Synthesis of Novel Functionalized Selenium Nanoparticles by *Lactobacillus casei* ATCC 393 and its Protective Effects on Intestinal Barrier Dysfunction Caused by Enterotoxigenic *Escherichia*
*coli* K88. Front. Microbiol..

[28] Nagy G., Pinczes G., Pinter G., Pocsi I., Prokisch J., Banfalvi G. (2016). In Situ Electron Microscopy of Lactomicroselenium Particles in Probiotic Bacteria. Int. J. Mol. Sci..

[29] Alam H., Khatoon N., Khan M.A., Husain S.A., Saravanan M., Sardar M. (2020). Synthesis of Selenium Nanoparticles Using Probiotic Bacteria *Lactobacillus acidophilus* and their Enhanced Antimicrobial Activity Against Resistant Bacteria. J. Clust. Sci..

[30] Pieniz S., Andreazza R., Mann M.B., Camargo F., Brandelli A. (2017). Bioaccumulation and Distribution of Selenium in Enterococcus durans. J. Trace Elem. Med. Biol..

[31] Martínez F.G., Moreno-Martin G., Pescuma M., Madrid-Albarrán Y., Mozzi F. (2020). Biotransformation of Selenium by Lactic Acid Bacteria: Formation of Seleno-Nanoparticles and Seleno-Amino Acids. Front. Bioeng. Biotechnol..

[32] Yang J., Li Y., Zhang L., Fan M., Wei X. (2017). Response Surface Design for Accumulation of Selenium by Different Lactic Acid Bacteria. 3 Biotech.

[33] Eszenyi P., Sztrik A., Babka B., Prokisch J. (2011). Elemental, Nano-Sized (100–500 nm) Selenium Production by Probiotic Lactic Acid Bacteria. Int. J. Biosci. Biochem. Bioinformatics.

[34] Hosnedlova B., Kepinska M., Skalickova S., Fernandez C., Ruttkay-Nedecky B., Peng Q., Baron M., Melcova M., Opatrilova R., Zidkova J. (2018). Nano-Selenium and its Nanomedicine Applications: A Critical Review. Int. J. Nanomed..

[35] Behne D., Wolters W. (1983). Distribution of Selenium and Glutathione Peroxidase in the Rat. J. Nutr..

[36] Gu Q.P., Xia Y.M., Ha P.C., Butler J.A., Whanger P.D. (1998). Distribution of Selenium Between Plasma Fractions in Guinea Pigs and Humans with Various Intakes of Dietary Selenium. J. Trace Elem. Med. Biol..

[37] Arteel G.E., Sies H. (2001). The Biochemistry of Selenium and the Glutathione System. Environ. Toxicol. Pharmacol..

[38] Rýdlová M., Růnová K., Száková J., Fučíková A., Hakenová A., Mlejnek P., Zídek V., Tremlová J., Mestek O., Kaňa A. (2017). The Response of Macro- and Micronutrient Nutrient Status and Biochemical Processes in Rats Fed on a Diet with Selenium-Enriched Defatted Rapeseed and/or Vitamin E Supplementation. BioMed Res. Int..

[39] Fujihara T., Orden E.A. (2014). The Effect of Dietary Vitamin E Level on Selenium Status in Rats. J. Anim. Physiol. Anim. Nutr..

[40] Arnaud J., van Dael P., Michalke B. (2018). Selenium Interactions with Other Trace Elements, with Nutrients (And Drugs) in Humans. Selenium. Molecular and Integrative Toxicology.

[41] Benstoem C., Goetzenich A., Kraemer S., Borosch S., Manzanares W., Hardy G., Stoppe C. (2015). Selenium and its Supplementation in Cardiovascular Disease—What do We Know?. Nutrients.

[42] Kurek E., Ruszczynska A., Wojciechowski M., Czauderna M., Bulska E. (2009). Study on Speciation of Selenium in Animal Tissues Using High Performance Liquid Chromatography with on-line Detection by Liquid Coupled Plasma Mass Spectrometry. Chem. Anal..

[43] Qin S., Gao J., Huang K. (2007). Effects of Different Selenium Sources on Tissue Selenium Concentrations, Blood gsh-px Activities and Plasma Interleukin Levels in Finishing Lambs. Biol. Trace Elem. Res..

[44] Han X.J., Qin P., Li W.X., Ma Q.G., Ji C., Zhang J.Y., Zhao L.H. (2017). Effect of Sodium Selenite and Selenium Yeast on Performance, Egg Quality, Antioxidant Capacity, and Selenium Deposition of Laying Hens. Poult. Sci..

[45] Sobeková A., Holovská K., Lenártová V., Holovská K., Javorský P., Boldižárová K., Grešáková L., Leng L. (2006). Effects of Feed Supplemented with Selenite or Se-Yeast on Antioxidant Enzyme Activities in Lamb Tissues. J. Anim. Feed Sci..

[46] Zhou Y., Zhu H., Qi Y., Wu C., Zhang J., Shao L., Tan J., Chen D. (2020). Absorption and Distribution of Selenium Following Oral Administration of Selenium-Enriched Bifidobacterium Longum DD98, Selenized Yeast, or Sodium Selenite in Rats. Biol. Trace Elem. Res..

[47] Zhang B., Piao J., Gu L. (2002). Effects of selenium-enriched garlic on blood lipids and lipid peroxidation in Experimental Hyperlipidemic Rats. Wei Sheng Yan Jiu.

[48] Marounek M., Dokoupilová A., Volek Z., Hoza I. (2009). Quality of Meat and Selenium Content in Tissues of Rabbits Fed Diets Supplemented with Sodium Selenite, Selenized Yeast and Selenized Algae. World Rabbit Sci..

[49] Zhao L., Sun L.H., Huang J.Q., Briens M., Qi D.S., Xu S.W., Lei X.G. (2017). A Novel Organic Selenium Compound Exerts Unique Regulation of Selenium Speciation, Selenogenome, and Selenoproteins in Broiler Chicks. J. Nutr..

[50] Misra S., Peak D., Chen N., Hamilton C., Niyogi S. (2012). Tissue-Specific Accumulation and Speciation of Selenium in Rainbow Trout (*Oncorhynchus mykiss*) Exposed to Elevated Dietary Selenomethionine. Comp. Biochem. Physiol. C Toxicol. Pharmacol..

[51] Sánchez-Martínez M., Pérez-Corona T., Martínez-Villaluenga C., Frías J., Peñas E., Porres J.M., Urbano G., Cámara C., Madrid Y. (2014). Synthesis of 77Se-Methylselenocysteine when Preparing Sauerkraut in the Presence of 77Se-Selenite. Metabolic Transformation of 77Se-Methylselenocysteine in Wistar Rats Determined by LC–IDA–ICP–MS. Anal. Bioanal. Chem..

[52] Juniper D.T., Phipps R.H., Ramos-Morales E., Bertin G. (2008). Selenium Persistency and Speciation in the Tissues of Lambs Following the Withdrawal of Dietary High-Dose Selenium-Enriched Yeast. Animal.

[53] Gawor A., Ruszczynska A., Czauderna M., Bulska E. (2020). Determination of Selenium Species in Muscle, Heart, and Liver Tissues of Lambs Using Mass Spectrometry Methods. Animals.

[54] Cheng Y., Huang Y., Liu K., Pan S., Qin Z., Wu T., Xu X. (2021). Cardamine Hupingshanensis Aqueous Extract Improves Intestinal Redox Status and Gut Microbiota in Se-deficient Rats. J. Sci. Food Agric..

[55] Hrdina J., Banning A., Kipp A., Loh G., Blaut M., Brigelius-Flohé R. (2009). The Gastrointestinal Microbiota Affects the Selenium Status and Selenoprotein Expression in Mice. J. Nutr. Biochem..

[56] Kasaikina M.V., Kravtsova M.A., Lee B.C., Seravalli J., Peterson D.A., Walter J., Legge R., Benson A.K., Hatfield D.L., Gladyshev V.N. (2011). Dietary Selenium Affects Host Selenoproteome Expression by Influencing the Gut Microbiota. FASEB J..

[57] Takahashi K., Suzuki N., Ogra Y. (2020). Effect of Gut Microflora on Nutritional Availability of Selenium. Food Chem..

[58] Zhai Q., Cen S., Li P., Tian F., Zhao J., Zhang H., Chen W. (2018). Effects of Dietary Selenium Supplementation on Intestinal Barrier and Immune Responses Associated with its Modulation of Gut Microbiota. Environ. Sci. Technol. Lett..

[59] Zhu H., Zhou Y., Qi Y., Ji R., Zhang J., Qian Z., Wu C., Tan J., Shao L., Chen D. (2019). Preparation and Characterization of Selenium Enriched-*Bifidobacterium Longum* DD98 and its Repairing Effects on Antibiotic-Induced Intestinal Dysbacteriosis in Mice. Food Funct..

[60] Yang S., Li L., Yu L., Sun L., Li K., Tong C., Xu W., Cui G., Long M., Li P. (2020). Selenium-Enriched Yeast Reduces Caecal Pathological Injuries and Intervenes Changes of the Diversity of Caecal Microbiota Caused by Ochratoxin-A in broilers. Food Chem. Toxicol..

[61] Gangadoo S., Dinev I., Chapman J., Hughes R.J., Van T.T.H., Moore R.J., Stanley D. (2018). Selenium Nanoparticles in Poultry Feed Modify Gut Microbiota and Increase Abundance *Faecalibacterium Prausnitzii*. Appl. Microbiol. Biotechnol..

[62] Gangadoo S., Bauer B.W., Bajagai Y.S., Van T.T.H., Moore R.J., Stanley D. (2019). In Vitro Growth of Gut Microbiota with Selenium Nanoparticles. Anim. Nutr..

[63] Zhang Y., Xiao L., Hao Q., Li X., Liu F. (2020). Ferrihydrite Reduction Exclusively Stimulated Hydrogen Production by *Clostridium* with Community Metabolic Pathway Bifurcation. ACS Sustain. Chem. Eng..

[64] List C., Hosseini Z., Lederballe Meibom K., Hatzimanikatis V., Bernier-Latmani R. (2019). Impact of Iron Reduction on the Metabolism of *Clostridium Acetobutylicum*. Environ. Microbiol..

[65] Butel M.J., Roland N., Hibert A., Popot F., Favre A., Tessedre A.C., Bensaada M., Rimbault A., Szylit O. (1998). Clostridial Pathogenicity in Experimental Necrotising Enterocolitis in Gnotobiotic Quails and Protective Role of Bifidobacteria. J. Med. Microbiol..

[66] Rada V., Nevoral J., Trojanová I., Tománková E., Smehilová M., Killer J. (2008). Growth of Infant Faecal Bifidobacteria and Clostridia on Prebiotic Oligosaccharides in in vitro Conditions. Anaerobe.

[67] Zare H., Vahidi H., Owlia P., Khujin M.H., Khamisabadi A. (2017). Yeast Enriched with Selenium: A Promising Source of Selenomethionine and Seleno-Proteins. Trends Pept. Protein Sci..

